# Comparison of methods for the detection of in vitro synergy in multidrug-resistant gram-negative bacteria

**DOI:** 10.1186/s12866-020-01756-0

**Published:** 2020-04-16

**Authors:** Juliana Januario Gaudereto, Lauro Vieira Perdigão Neto, Gleice Cristina Leite, Evelyn Patricia Sanchez Espinoza, Roberta Cristina Ruedas Martins, Gladys Villas Boa Prado, Flavia Rossi, Thais Guimarães, Anna Sara Levin, Silvia Figueiredo Costa

**Affiliations:** 1 Laboratório de Investigação Médica 49 - LIM-49, Instituto de Medicina Tropical, Hospital das Clínicas, Faculdade de Medicina, Universidade de São Paulo, Avenida Doutor Enéas de Carvalho Aguiar, 470, São Paulo, SP 05403-000 Brazil; 2 Divisão de Laboratório Central - Serviço de Microbiologia Clínica, Hospital das Clínicas, Faculdade de Medicina, Universidade de São Paulo, São Paulo, SP Brazil

**Keywords:** Synergy, Time-kill, Disk approximation, MIC:MIC ratio, Gram-negative, Multidrug-resistant

## Abstract

**Background:**

The use of combined antibiotic therapy has become an option for infections caused by multidrug-resistant (MDR) bacteria. The time-kill (TK) assay is considered the gold standard method for the evaluation of in vitro synergy, but it is a time-consuming and expensive method.

The purpose of this study was to evaluate two methods for testing in vitro antimicrobial combinations: the disk diffusion method through disk approximation (DA) and the agar gradient diffusion method via the MIC:MIC ratio. The TK assay was included as the gold standard. MDR Gram-negative clinical isolates (*n* = 62; 28 *Pseudomonas aeruginosa*, 20 *Acinetobacter baumannii*, and 14 *Serratia marcescens*) were submitted to TK, DA, and MIC:MIC ratio synergy methods.

**Results:**

Overall, the agreement between the DA and TK assays ranged from 20 to 93%. The isolates of *A. baumannii* showed variable results of synergism according to TK, and the calculated agreement was statistically significant in this species against fosfomycin with meropenem including colistin-resistant isolates. The MIC:MIC ratiometric agreed from 35 to 71% with TK assays. The kappa test showed good agreement for the combination of colistin with amikacin (K = 0.58; *P* = 0.04) among the colistin-resistant *A. baumannii* isolates.

**Conclusions:**

The DA and MIC:MIC ratiometric methods are easier to perform and might be a more viable tool for clinical microbiology laboratories.

## Background

Infections with multidrug-resistant (MDR) bacteria have increased dramatically over the last decade and are a major global challenge [1]. The development of new antimicrobial agents has not kept up with the emergence of new mechanisms of antibiotic resistance [2]. Moreover, inappropriate initial antimicrobial therapy against MDR pathogens is associated with adverse outcomes. Therefore, reducing the turnaround time while testing antimicrobial efficacy including combinations of antibiotics can lead to significant reductions in patient morbidity, mortality, and cost.

Combined antimicrobial therapy is a promising strategy for treating infections caused by MDR pathogens and can further extend antimicrobial lifespan and minimize the evolution of resistance [3, 4]. However, despite the importance of in vitro testing, methods that are accessible to clinical microbiology laboratories for testing synergism in a clinically actionable period are not available.

Existing methods have several disadvantages including the time-kill assay (TK), which—although considered the gold standard for synergism evaluation—is very time-consuming and requires high technical skills [5, 6]. The disk and epsilometer tape diffusion methods using commercially available materials are less technically demanding than the TK assay but require further validation. In this study, we evaluated two in vitro antimicrobial combination methods as alternatives to the TK method in clinical microbiology laboratories.

## Results

All *A. baumannii* isolates were resistant to meropenem (MIC ranging from 16 to 128 μg/mL) and fosfomycin (MIC ranged from 64 to 128 μg/mL); 19/20 (95%) were resistant to amikacin. *Pseudomonas aeruginosa* isolates were susceptible to colistin and resistant to meropenem (MIC ranged from 16 to 512 μg/mL). Resistance to amikacin was observed in 64% (18/28) of the *Pseudomonas aeruginosa* isolates. Resistance to carbapenems was found in 86% (12/14) of *S. marcescens* isolates, and 64% (9/14) were resistant to amikacin. The results of sequence type (ST) and antimicrobial resistance genes from each isolate are summarized in Table 1.

**Table 1:** Sequence type according to MLST Oxford and antimicrobial resistance genes determined by PCR and WGS for 20 *A. baumannii,* 28 *P. aeruginosa,* and 14 *S. marcescens*

ID	Sequence Type	Antimicrobial resistance genes
***Acinetobacter baumannii***		
1	32	*bla*_OXA-51_,bla_IMP-1_, aacA4, aac(6′)-31, aadA1, fosA, aac(6′)Ib-cr, sul1
2	ND	*Bla*_Oxa-51_
3	15	*bla*_OXA-51_, aph(3′)-Via,fosA,fosX,
4	107	*bla*_OXA-51_, *bla*_OXA-143_,aadB, strA, strB,fosA, floR, sul2,
5	ND	*Bla*_Oxa-51_
6	15	*bla*_OXA-51_,*bla*_IMP_, fosA,fosX
7	ND	*bla*_OXA-51_, *bla*_IMP_
8	317	*bla*_OXA-117_, aph(3′)-Via, FosA,FosX
9	107	*bla*_OXA-51_,*bla*_OXA-143_, aph(3′)-Via, aadB, fosA, floR, sul2
10	ND	*bla*_OXA-51_,*bla*_OXA-143_,*bla*_IMP_
11	ND	*bla*_Oxa-51_
12	317	*bla*_OXA-51_, *bla*_OXA-23_, aph(3′)-Via, fosA,fosX,
13	107	*bla*_OXA-51_,*bla*_OXA-143_, aph(3′)-VIa, aadB, strA, strB, fosA, floR,sul2
14	79	*bla*_OXA-23_, *bla*_OXA-65_, *bla*_TEM-1_, aph(3′)-VIa, aadA1, strA, strB, fosA, sul2,dfrA1
15	79	*bla*_OXA-23_, *bla*_OXA-117_, *bla*_TEM-1_, aph(3′)-VIa, aadA1, strA, strB, fosA, sul2,dfrA1
16	836	*bla*_OXA-117_,*bla*_TEM-1_, aph(3′)-VIa, aadA1, strA, strB, fosA, dfrA1
17	79	*bla*_OXA-23_, *bla*_OXA-65_, *bla*_TEM-1_, aph(3′)-VIa, aadA1, strA, strB, fosA,, sul2,dfrA1
18	79	*bla*_OXA-51_, *bla*_OXA-23_, *bla*_TEM-1_, aph(3′)-VIa, aadA1, strA, strB, fosA, sul2,dfrA1
19	317	*bla*_OXA-51_, *bla*_OXA-23_,fosA,
20	79	*bla*_OXA-117_, *bla*_TEM-1_, aadA1, strA, strB, fosA, floR, sul2,dfrA1
***Pseudomonas aeruginosa***		
1	277	*bla*_*KPC*_*, bla*_OXA-50_, *bla*_PAO_,*bla*_SPM-1_,*bla*_OXA-56_, *aph(3′)-IIb*,*aadA7*, *rmtD*, aacA4, *fosA*, *aac(6′)Ib-cr*, cmx, *catB7*, *sul1*
2	ND	*bla*_KPC,_*bla*_SPM_
3	ND	*bla*_KPC,_*bla*_SPM_
4	ND	*bla*_KPC,_*bla*_SPM_
5	1853	*bla*_*KPC*_*, bla*_OXA-50_, *bla*_PAO,_*aph(3′)-IIb*, *fosA*, *catB7*
6	ND	*bla*_KPC,_*bla*_SPM_
7	ND	*bla*_SPM_
8	ND	*bla*_SPM_
9	ND	–
10	ND	*bla*_SPM_
11	277	*bla*_OXA-50_,*bla*_PAO_,*bla*_SPM-1_,*bla*_OXA-56_,*aph(3′)-Iib*, *aadA7*, *aacA4*, *aac(6′)Ib-cr*, *cmx*,*catB8*
12	ND	*bla*_SPM_
13	ND	*bla*_SPM_
14	277	*bla*_*KPC*_*, bla*_OXA-50_,*bla*_PAO_,*bla*_SPM-1_,*bla*_OXA-56_, *aph(3′)-IIb*,*aadA7*, rmtD, *aacA4*, *fosA*, *aac(6′)Ib-cr*, *cmx*, *catB7*, *sul1*
15	ND	–
16	ND	–
17	ND	*bla*_SPM_
18	ND	*bla*_SPM_
19	ND	*bla*_SPM_
20	277	*bla*_OXA-50_,*bla*_PAO_,*bla*_SPM-1_,*bla*_OXA-56_,*aph(3′)-IIb*,*aadA7*, *rmtD*, *aacA4*, fosA, *aac(6′)Ib-cr*, *catB7*, *sul1*
21	ND	*bla*_SPM_
22	ND	*bla*_SPM_
23	ND	–
24	ND	*bla*_SPM_
25	ND	*bla*_SPM_
26	ND	–
27	ND	*bla*_SPM_
28	ND	*bla*_SPM_
***Serratia marcescens***		
1	NA	*bla*_SRT-2_, *bla*_KPC-2_, *aph(3′)-*VIa, *aacA4*, *aac(6′)-Ic*, *ant(2″)-Ia*, *aac(6′)-Ib-cr*, *cat (pC194)*, *sul2*, *dfrA8*
2	NA	*bla*_SRT-2_, *bla*_KPC-2_, *aph(3′)-*VIa, *aacA4*, *aac(6′)-Ic*, *ant(2″)-Ia*,*aac(6′)-Ib-cr*,*sul2*,*dfrA8*
3	NA	*bla*_SRT-2_, *bla*_KPC-2_, *aph(3′)-*VIa, *aacA4*, *aac(6′)-Ic*, *ant(2″)-Ia*, *aac(6′)-Ib-cr*, *cat (pC194)*, *sul2*
4	NA	*bla*_SRT-2_, *bla*_KPC-2_,*aph(3′)-*VIa, *aacA4*, *aac(6′)-Ic*, *ant(2″)-Ia*, *aac(6′)-Ib-cr*,*sul2*, *dfrA8*
5	NA	*bla*_SRT-2_, *bla*_KPC-2_, *aph(3′)-*VIa, *aacA4*, *aac(6′)-Ic*, *ant(2″)-Ia*,*aac(6′)-Ib-cr*,*sul2*, *dfrA8*
6	NA	*bla*_SRT-2_, *bla*_KPC-2_, *aph(3′)-*VIa, *aacA4*,*aac(6′)-Ic*,*ant(2″)-Ia*,*aac(6′)-Ib-cr*,*sul2*, *dfrA8*
7	NA	*bla*_SRT-2_, *bla*_CTX-M-2_, *bla*_TEM-1A_, *bla*_OXA-101_, *aph(3′)-*VIa, *aadA6*, *aac(6′)-Ic*, *ant(2″)-Ia*, *cat (pC194)*, *cmx*, *sul1*, *sul1*, *dfrA22*
8	NA	*bla*_SRT-2_, *bla*_KPC-2_, *aph(3′)-*VIa, *aacA4*, *aac(6′)-Ic*,*ant(2″)-Ia*, *aac(6′)-Ib-cr*, *cat (pC194)*, *sul2*, *dfrA8*
9	NA	*bla*_SRT-2_, *aacA4*, *aac(6′)-Ic*, *ant(2″)-Ia*, *aac(6′)-Ib-cr*, *sul2*, *dfrA8*
10	NA	*bla*_SRT-2_, *bla*_KPC-2_, *aacA4*, *aac(6′)-Ic*, *ant(2″)-Ia*, *aac(6′)-Ib-cr*, *sul2*, *dfrA8*
11	NA	*bla*_SRT-2_, *bla*_KPC-2_, *bla*_SHV-5_, *aacA4*, *aac(6′)-Ic*, *ant(2″)-Ia*, *sul2*, *dfrA1*
12	NA	–
13	NA	*bla*_SRT-2_, *bla*_OXA-101_, *aph(3′)-*VIa, *aadA6*, *aac(6′)-Ic*, *ant(2″)-Ia*, *cmx*,*sul1*, *dfrA1*
14	NA	*bla*_SRT-2_, *bla*_KPC-2_, *bla*_SHV-5_, *aacA4*, *aac(6′)-Ic*, *ant(2″)-Ia*, *aac(6′)-Ib-cr*, *sul2*

*ID* Isolates; *ND* not WGS; *NA* not applicable

The TK assay showed that *A. baumannii* isolates had synergistic effects with colistin combinations. Agreement with the DA method was 86% among the seven colistin-resistant isolates except for the combination of colistin with amikacin, which showed an agreement of 14%. The combination of fosfomycin with meropenem showed 50% synergistic effects via time-kill for *A. baumannii*. The agreement between DA and TK was considered good (k = 0.60; *P* = 0.003), and the correlation was good among the seven colistin-resistant isolates (k = 0.72; *P* = 0.02) (Table 2).

**Table 2:** Concordance analysis data among disk approximation and MIC:MIC ratio methods with time-kill assay according to antimicrobial combinations tested for *A. baumannii, P. aeruginosa,* and *S. marcescens*

Drugs combination/Species	Disk approximation and TK		MIC:MIC ratio and TK	
***Acinetobacter baumannii*(*n*** **= 20)**	Concordance (%)	Kappa/*p* value	Concordance (%)	Kappa/p value
Colistin with meropenem	30	0.0/0.500	40	0.0
Colistin with amikacin	20	0.0/0.333	35	0.04/0.25
Colistin with fosfomycin	30	0.0/0.500	NA	NA
Fosfomycin with meropenem	80	0.60/0.003	NA	NA
Fosfomycin with gentamycin	75	−0.0/0.696	NA	NA
***Pseudomonas aeruginosa*(*n*** **= 28)**				
Colistin with meropenem	57	0.0	57	0.0
Colistin with amikacin	93	0.0	68	0.0
Meropenem with amikacin	64	0.0	NA	NA
***Serratia marcescens*(*n*** **= 14)**				
Colistin with meropenem	64	−0.12/0.744	NA	NA
Colistin with amikacin	93	0.0	NA	NA
Ertapenem with meropenem	86	0.0/0.500	71	0.25/0.081

*NA* Not applicable

The TK assay for *P. aeruginosa* isolates showed a synergistic effect for the combination of colistin + meropenem 43% (12/28) and meropenem + amikacin 36% (10/28). No synergistic effects were seen via the DA method. Agreement with TK for colistin with meropenem, colistin with amikacin, and meropenem with amikacin was 57, 93, and 64% respectively. The kappa test showed poor agreement for all of these combinations (Table 2).

The combination of colistin with meropenem had synergy against only one isolate of *S. marcescens* using the TK assay. Moreover, this isolate did not have a synergistic effect by DA. The DA and TK methods were concordant in 64, 93, and 86% with TK for colistin with meropenem, colistin with amikacin, and ertapenem with meropenem, respectively. The DA method showed poor agreement with the TK assay via the kappa test (Table 2). Overall, the agreement between the MIC:MIC ratio method and TK assays ranged from 35 to 71% (Table 2). The kappa test agreed well with colistin/amikacin (K = 0.58; *P* = 0.04) among the seven colistin-resistant *A. baumannii* isolates.

The MIC:MIC ratio method did not identify synergistic effects for *P. aeruginosa* isolates, and the Kappa results were not statistically significant. Synergistic effects were observed for one *S. marcescens* isolate in both DA and MIC:MIC methods; this isolate showed no synergistic effect in 0.5 × MIC (sub-inhibitory concentration) by TK assay. The agreement between the TK and MIC:MIC ratiometric methods was poor for the combination of ertapenem with meropenem. No antagonistic effects were noted.

## Discussion

Multidrug-resistant Gram-negative strains such as *A. baumannii*, *P. aeruginosa*, and *S. marcescens* are commonly studied due to their major role as nosocomial pathogens with frequent development of multidrug resistance [7–13]. The treatment of infections caused by these microorganisms based on identification of resistance mechanisms and drug combinations is usually more effective than empirical treatment [1, 4]. Synergy testing can also correlate to a particular resistance mechanism [8, 14]. Such correlations might help predict the synergism for a particular antimicrobial combination for treatment. Thus, determining the molecular mechanisms can improve therapeutic outcomes.

While the rapid detection of resistance mechanisms is performed in several healthcare centers, antimicrobial interaction tests are still not available due to the challenges associated with routine combination tests. The method described here can be useful in selecting the proper combinations of antibiotics. Here, accurate and prompt treatment has an important effect on the reduction of morbidity, mortality, and costs.

Several methods have been assessed to evaluate the synergistic activity of two or more antimicrobial agents [5, 6, 15–18]. As the gold standard, the TK method yields high concordance between various studies because it produces dynamic and longitudinal information about bacterial death, which is not provided by other methods. However, the TK method is a complex technique that is difficult to perform in routine laboratories. On the other hand, antimicrobial disk methods are affordable and simple, but there are limited data evaluating the synergism of this technique in vitro with controversial results [15, 17, 18].

In the present study, the agreement between the TK assay and the DA method was higher among colistin-resistant *A. baumannii* isolates when colistin was combined with meropenem or Fosfomycin similar to other studies [7, 9–14]. The synergistic effect of fosfomycin with carbapenem could be explained by the resistance profile of the strains evaluated—most *A. baumannii* isolates carried carbapenemase genes (*bla*_OXA-23,_*bla*_OXA-143_, and *fos*A), which confer resistance to fosfomycin. Both classes of antimicrobials act at different sites of the bacterial cell wall and inhibit cell wall synthesis [7, 9]. Perdigão-Neto et al. [19] also demonstrated that fosfomycin is a promising drug—particularly in combination with meropenem for the treatment of infections due to pan-resistant Gram-negative bacteria.

Nevertheless, the in vitro synergy effect of colistin against *S. marcescens* is controversial [11, 16]. The synergistic effect appears lower for combinations with colistin among *S. marcescens* isolates and species that exhibit intrinsic resistance to polymyxins [14]. In our study, we found a lower synergistic effect against *S. marcescens* isolates in colistin combinations consistent with Nastro [11] who evaluated colistin with rifampicin against colistin-resistant *A. baumannii*, *K. pneumoniae*, and *S. marcescens* isolates by E-test/agar dilution and TK assays. This work noted synergy for all isolates except two of five *S. marcescens* isolates. Thus, it seems that combinations using colistin are not useful for treating Serratia infections.

Few studies have compared the MIC:MIC ratio and the TK assay against a large collection of MDR bacteria [6, 20–22]. One of the largest studies [6] compared three E-test methods with TK against 31 KPC-producing *Klebsiella* isolates. The MIC:MIC ratio showed a better correlation with the TK assay: concordance of 80.6% and a significant Kappa value of 0.59 (*P* < 0.001). In our study, the agreement was statistically significant only for the combination of colistin with amikacin (K = 0.58; *P* = 0.04) among the colistin-resistant *A. baumannii* isolates.

The agreement between MIC:MIC ratio and TK assays ranged from 35 to 71%, which is similar to those described by Chachanidze et al. [23] who compared the results of TK with MIC:MIC ratio for 31 fluoroquinolone-resistant *P. aeruginosa* isolates. These authors evaluated a combination of levofloxacin and piperacillin/tazobactam and found 77% agreement between the methods. Some studies have shown clinical application of synergism [19, 24]. Perdigão Neto et al. showed better clinical outcomes in Gram-negative infections treated with combined therapy with known in vitro synergism [19]. In addition, some studies have shown the benefit of combined therapy despite the resistance of the microorganism to some antimicrobials in the scheme [24].

Synergism has also been described using DA and MIC:MIC methods against Gram-positive organisms—especially *S. aureus* [25–27]; these are likely useful methods against Gram-positive organisms. Unfortunately, for logistical reasons, we could not evaluate in vitro synergy against Gram-positive standard organisms. This is an important limitation of our study. We evaluated *A. baumannii* belonging to international STs such as ST15 and ST297 [28, 29] as well as *S. marcescens* carrying KPC—the most frequent carbapenemase described for this organism [1, 2]. Thus, our findings can be useful internationally.

There are some important limitations to this study. First, we tested a relatively small number of isolates from only one hospital and the *P. aeruginosa* isolates belong to the endemic clone ST277 isolated mainly in Brazil [30]. However, the isolates evaluated were identified during an 11-year period and have been well-characterized (phenotypically and genotypically). Therefore, our findings indicate that DA and MIC:MIC ratio methods can be useful to help infectious disease clinicians handle infections caused by carbapenem-resistant organisms. DA and MIC:MIC methods can highlight the in vitro synergy and avoid combination therapies that will increase cost and side effects.

## Conclusions

We found that the DA method has good agreement with the TK assay for Fosfomycin/meropenem combinations against colistin-resistant *A. baumannii* isolates carrying carbapenemases and *fos*A genes. The feasibility of the DA method depends on the bacterial resistance mechanism. The DA and MIC:MIC ratio methods are easy to perform and are suitable for the screening of synergy in clinical microbiology laboratories. Further studies are needed to evaluate these methods against a large collection of organisms including Gram-positive bacteria.

## Methods

### Bacterial isolates

We selected 62 clinical isolates of MDR Gram-negative bacteria studied previously [3]. This cohort included 20 *A. baumannii* isolates identified from 2002 to 2012; 28 *P. aeruginosa* isolates from 2011 to 2013; and 14 *S. marcescens* isolates from 2010 to 2013. All samples were collected from patients at the Hospital das Clínicas da Universidade de São Paulo (HC-FMUSP). Identification was performed using an automated Vitek 2 system (BioMérieux, Hazelwood, MO). All non-fermenting isolates and 86% of Enterobacteria were carbapenem-resistant; seven isolates of *A. baumannii* were colistin-resistant.

### *G*enotypic characterization

Carbapenemases genes (*bla*_OXA-23-like_, *bla*_OXA-51-like_, *bla*_OXA-58-like_, *bla*_OXA-24-like_, *bla*_IMP_, *bla*_SPM_, *bla*_VIM_, *bla*_SIM_, *bla*_NDM_, *bla*_OXA-143-like_, and *bla*_KPC_) were investigated by PCR [31–33], and the other genes were studied by whole-genome sequence (WGS). Thirty-four isolates (16 *A. baumannii*, 13 *S. marcescens*, and 5 *P. aeruginosa*) were characterized by WGS using MiSeqIllumina™ technology. The files were analyzed by VelvetOptimizer v.2.2.5 software (Victorian Bioinformatics Consortium, Australia). Genome annotation was performed using Prokka [34]. The resistance genes were investigated using Resfinder (https://cge.cbs.dtu.dk). The sequence type (ST) was determined by MLSTfinder (Multilocus Sequence Typing) [35].

### Antimicrobial sensitivity test

The minimal inhibitory concentrations (MIC) of colistin, meropenem (USP Reference Standard, Rockville, MD, USA), gentamicin, amikacin, and ertapenem (Sigma - Aldrich, St Louis, MO, USA) were determined via the broth microdilution method, and fosfomycin was determined (Sigma - Aldrich, St Louis, MO, USA) via the gold-standard agar dilution method. The assays were performed in duplicate on alternate days as recommended by the Clinical and Laboratory Standards Institute (CLSI) [36]; samples were quality control tested with *P. aeruginosa* ATCC 27853 and *E. coli* ATCC 25922 strains. CLSI-recommended breakpoints were used for all antimicrobials except for colistin and fosfomycin for which European Committee on Antimicrobial Susceptibility Testing (EUCAST) breakpoints were used including the breakpoints for fosfomycin in *Enterobacteriaceae* for the categorization of the *A. baumannii* isolates [37].

### Synergy tests

To identify synergistic effects, the TK, disk approximation, and MIC:MIC ratio methods were performed in duplicate. Each antimicrobial combination was chosen as previously described in the literature and the treatment options available in our hospital. For the MIC:MIC ratio, we tested the susceptibility of non-fermenting Gram-negative isolates against colistin with meropenem and colistin with amikacin combination as well as the susceptibility of *Enterobacteria* isolates against ertapenem with meropenem.

#### Time-kill method

The TK method was performed as previously described [38]. Antimicrobials were tested alone and in combination with concentrations ranging from 1× to 0.5× MIC. Control experiments without antimicrobial agents were conducted simultaneously with the TK assay. The vials containing cation-adjusted Mueller-Hinton broth, antimicrobials, and the tested organisms at an initial density of 10^6^ CFU/ml (10 ml volume) were incubated at 35 ± 2 °C in ambient air. Aliquots were removed at 0, 2, 4, 6, and 24 h and serially diluted in 0.9% sodium chloride solution and plated on Mueller-Hinton agar plates for viable-colony counting. The synergy effect was defined as a ≥ 2 log10 CFU/ml reduction in colony counting when compared to the most active single agent after incubation for 24 h. The antagonism was defined by an increase of ≥2 log10 CFU/ml in the combination versus the most active single agent. The no difference (ND) effect was established as an increase or decrease of < 2 log10 in colony counting with an antibiotic combination versus individual antimicrobials [38].

#### Disk approximation (DA)

Commercial disks were purchased from Oxoid® (Basingstoke, UK) including colistin (10 μg), amikacin (30 μg), gentamicin (10 μg), meropenem (10 μg), ertapenem (10 μg), fosfomycin (200 μg), and gentamicin (10 μg). These were placed 5 mm apart on 150-mm diameter Mueller-Hinton agar plates cultured with organisms adjusted to the 0.5 McFarland standard and incubated at 35 ± 2 °C for 16 to 18 h [39]. Synergism was defined by inhibition zone bridging. Antagonism was indicated by truncation of the inhibition zone at the junction of the antimicrobials; ND was defined as the formation of two independent circles around the antibiotic disks [15].

#### MIC:MIC ratio

The MIC was initially determined using strips impregnated with colistin (bioMérieux, France), amikacin, meropenem (Thermo Fisher Scientific, Basingstoke, UK), and ertapenem (Liofilchem, Italy) at increasing concentrations. For the synergism testing, one test strip was placed on the inoculated MHA plate. After 1 h at room temperature, the agar was marked adjacent to the previously determined MIC of the agent, and the tape was replaced. The second strip was then placed over the imprint of the previous strip such that the mark on the agar corresponds to the MIC of the second agent [20]. The highest value was considered for isolates in which the MIC exceeded the value of the strip concentration [6]. The resulting ellipse of inhibition was checked after 18–20 h at 35 ± 2 °C and the Fractional Inhibitory Concentration Index (ΣFIC) was calculated and interpreted using the following criteria: synergism, ΣFIC ≤0.5; antagonism, ΣFIC> 4; and indifferent, ΣFIC> 0.5–4 [6, 15, 20, 31–40].

### Analysis of the results of synergy tests

The Kappa statistical test was performed using STATA software (College Station, TX, USA) version 13. The results of the DA and MIC:MIC ratio were compared to the TK results to establish the gold standard. The concentration of 1× MIC was chosen for comparison of the TK with the MIC:MIC ratio because this concentration used an epsilometric test [6]. The results were interpreted as poor agreement when k < 0.40; good agreement when k = 0.40–0.75; and very good agreement when k > 0.75 [40]. *P-*values < 0.05 was considered statistically significant. The correlation was calculated by a ratio of concordant responses among the evaluated methods.

## Data Availability

The datasets used and/or analysed during the current study available from the corresponding author on reasonable request.

## Abbreviations

CLSI: Clinical and Laboratory Standards Institute
DA: Disk approximation
EUCAST: European Committee on Antimicrobial Susceptibility Testing
FIC: Fractional inhibitory concentration
MDR: Multidrug-resistant
MIC: Minimal inhibitory concentration
TK: Time-kill; WGS: whole-genome sequence
